# Involvement of ERK Phosphorylation of Trigeminal Spinal Subnucleus Caudalis Neurons in Thermal Hypersensitivity in Rats with Infraorbital Nerve Injury

**DOI:** 10.1371/journal.pone.0057278

**Published:** 2013-02-22

**Authors:** Ikuko Suzuki, Yoshiyuki Tsuboi, Masamichi Shinoda, Kazuo Shibuta, Kuniya Honda, Ayano Katagiri, Masaaki Kiyomoto, Barry J. Sessle, Shingo Matsuura, Kinuyo Ohara, Kentaro Urata, Koichi Iwata

**Affiliations:** 1 Department of Physiology, Nihon University School of Dentistry, Chiyoda-ku Tokyo, Japan; 2 Department of Oral and Maxillofacial Surgery, Nihon University School of Dentistry, Chiyoda-ku Tokyo, Japan; 3 Department of Oral Physiology, Faculty of Dentistry, University of Toronto, Toronto, Ontario, Canada; 4 Department of Endodontics, Nihon University School of Dentistry, Chiyoda-ku Tokyo, Japan; 5 Department of Prosthodontics, Nihon University School of Dentistry, Chiyoda-ku Tokyo, Japan; 6 Division of Functional Morphology, Dental Research Center, Nihon University School of Dentistry, Tokyo, Japan; 7 Division of Applied System Neuroscience Advanced Medical Research Center, Nihon University Graduate School of Medical Science, Tokyo, Japan; Rutgers University, United States of America

## Abstract

To evaluate the involvement of the mitogen-activated protein kinase (MAPK) cascade in orofacial neuropathic pain mechanisms, this study assessed nocifensive behavior evoked by mechanical or thermal stimulation of the whisker pad skin, phosphorylation of extracellular signal-regulated kinase (ERK) in trigeminal spinal subnucleus caudalis (Vc) neurons, and Vc neuronal responses to mechanical or thermal stimulation of the whisker pad skin in rats with the chronic constriction nerve injury of the infraorbital nerve (ION-CCI). The mechanical and thermal nocifensive behavior was significantly enhanced on the side ipsilateral to the ION-CCI compared to the contralateral whisker pad or sham rats. ION-CCI rats had an increased number of phosphorylated ERK immunoreactive (pERK-IR) cells which also manifested NeuN-IR but not GFAP-IR and Iba1-IR, and were significantly more in ION-CCI rats compared with sham rats following noxious but not non-noxious mechanical stimulation. After intrathecal administration of the MEK1 inhibitor PD98059 in ION-CCI rats, the number of pERK-IR cells after noxious stimulation and the enhanced thermal nocifensive behavior but not the mechanical nocifensive behavior were significantly reduced in ION-CCI rats. The enhanced background activities, afterdischarges and responses of wide dynamic range neurons to noxious mechanical and thermal stimulation in ION-CCI rats were significantly depressed following i.t. administration of PD98059, whereas responses to non-noxious mechanical and thermal stimulation were not altered. The present findings suggest that pERK-IR neurons in the Vc play a pivotal role in the development of thermal hypersensitivity in the face following trigeminal nerve injury.

## Introduction

Peripheral nerve injury may result in neuropathic pain which is characterized by severe persistent pain in the areas innervated by the injured nerve [1], [2], [3], [4], [5]. Following orofacial surgical treatment such as third molar tooth extraction, dental pulpectomy or dental implantation, neuropathic pain sometimes occurs in the orofacial region and is difficult to diagnose and treat, and its underlying mechanisms are unclear [6], [7], [8], [9], [10], [11].

A variety of animal models with trigeminal nerve injury have been developed to evaluate mechanisms underlying orofacial neuropathic pain, and include chronic constriction injury of the infraorbital nerve (ION-CCI) and inferior alveolar nerve (IAN) transection [6], [11], [12], [13], [14]. Molecular changes and enhancement of neuronal excitability can be observed in trigeminal ganglion (TG) neurons [15], [16], [17] and trigeminal spinal subnucleus caudalis (Vc) neurons after infraorbital or cervical nerve injury [18], [19], [20]. Mitogen-activated protein (MAP) kinase is one of the important molecules involved in the intracellular transduction cascade resulting in neuronal excitability changes [21], [22], [23], [24]. Extracellular signal-regulated kinase (ERK), p38 and c-Jun N-terminal kinases in sensory neurons are known to have different functions in sensory processing [25], [26], [27]. In the trigeminal nociceptive system, ERK is phosphorylated in Vc and upper cervical spinal cord (C1/C2) neurons within 5 min after strong noxious stimulation of the orofacial region, and the number of phosphorylated ERK (pERK)-immunoreactive (IR) Vc neurons increases as noxious stimulus intensity is increased [28], [29], [30].

It has also been reported that pERK-IR neurons are somatotopically organized in the Vc and C1/C2 following capsaicin injection into restricted areas of the orofacial region [31]. These findings strongly suggest that the ERK phosphorylation in Vc neurons is involved in modulation of neuronal excitability following various noxious stimuli. However, the functional involvement of ERK phosphorylation in Vc neurons in trigeminal neuropathic pain states is not fully understood, but it is very important to clarify mechanisms underlying ERK phosphorylation following trigeminal nerve injury in order to understand orofacial neuropathic pain mechanisms. Therefore, the aim of the present study was to assess nocifensive behavior evoked by mechanical or thermal stimulation of the whisker pad skin, phosphorylation of ERK in Vc neurons, and Vc neuronal responses to mechanical or thermal stimulation of the whisker pad skin in ION-CCI rats.

## Materials and Methods

### Animals

One hundred fifty nine male Sprague Dawley rats weighing 300–400 g (ION-CCI: n = 120, sham operation: n = 39) were used in this study. The rats were housed under 12 h light/dark cycle conditions and had free access to food and water except during the test period. To minimize animal suffering, the number of animals used was based on the minimum required for statistically valid results. Behavioral and electrophysiological analysis were carried out in a blind manner. This study was approved by the Animal Experimentation Committee at the Nihon University. All surgery and animal care were conducted in accordance with the National Institutes of Health Guide for the Care and Use of Laboratory Animals and the guidelines for Institutional Animal Care, and the guidelines of the International Association for the Study of Pain.

### Infraorbital nerve constriction

Rats were initially anesthetized with pentobarbital sodium (50 mg/kg, i.p.). For ION ligation, rats were placed on a warm mat and a small incision was made on the buccal mucous membrane at buccal side of the upper molar and ION was exposed. The ION was loosely ligated with 4-0 chromic guts (Myco Medical Supplies Unc., NC) at two points of the nerve trunk and the wound was sutured after ION ligation. For sham-operated rats, the mucous membrane was cut and ION was exposed but not ligated, and the wound was sutured.

### Behavioral testing

As previously described in detail, rats were trained daily to stay in a plastic cage for 20 min, to protrude their perioral region including the whisker pad skin through a hole on the wall of the plastic cage for 5 min and to keep their snout protruding through a hole while mechanical, heat and cold stimulation were applied to whisker pad skin [11], [32]. Rats could escape freely from stimulation under this condition. The experimenter was kept blind to the experimental conditions.

Previously described approaches were also used to test mechanical or thermal sensitivity in these rats [11]. Mechanical sensitivity of the whisker pad skin was assessed by the use of von Frey filaments (North Coast Medical, Morgan Hill, Calif). The mechanical stimulation was applied to the whisker pad skin ipsilateral or contralaterally in ION-CCI rats or ipsilaterally in sham rats with von Frey filaments before and on days 1, 3, 7, 14 and 21 after ION-CCI. The head-withdrawal threshold to mechanical stimulation of the whisker pad skin was defined as the minimum pressure needed to evoke an escape more than 3 times of 5 stimuli. A cutoff of 60 g was established to prevent tissue damage. Heat sensitivity of the whisker pad skin was assessed using a radiant heat stimulator (Intercross-2000, Intercross, Tokyo, Japan) applied ipsilaterally or contralaterally in ION-CCI rats or ipsilaterally in sham rats. Animals were restrained in the plastic cage, and the head-withdrawal latency to radiant heat stimulation was manually recorded with a chronometer before and on days 1, 3, 7, 14 and 21 after ION-CCI or sham operation. A cutoff of 10 s was established to prevent tissue damage. Cold sensitivity of the whisker pad skin was assessed using 50 µl acetone applied ipsilaterally or contralaterally in ION-CCI rats or ipsilaterally in sham rats. The number of face scratches was counted for 1 min before and on days 1, 3, 7, 14 and 21 after ION-CCI or sham operation.

### Immunohistochemistries

Since neuropathic pain behavior to mechanical, heat and cold stimuli was obvious at 7 days after ION-CCI, immunohistochemical studies were conducted in the rats on day 7 after the ION-CCI, sham operation or naïve rats. pERK immunohistochemistry was conducted in ION-CCI or sham rats or in ION-CCI rats with i.t. infusion of MEK1 inhibitor PD98059 (EMD Biosciences, La Jolla, CA) or isotonic saline (as vehicle control) that had received low-intensity (6 g) or high-intensity (60 g) mechanical stimulation to the maxillary whisker pad skin (1 Hz, duration for 15 min) with von Frey filaments. Five min after cessation of the stimulation, the rats were perfused with isotonic saline followed by 4% paraformaldehyde (PFA) in 0.1 M phosphate buffer (pH 7.4, 1 L/kg). Labeling of astroglial and microglial cells was also conducted by using glial fibrillary acid protein (GFAP) and ionized calcium binding adaptor molecule 1 (Iba1) immunohistochemistries in ION-CCI rats with i.t. infusion of PD98059 or isotonic saline; rats were anesthetized with pentobarbital sodium (50 mg/kg, i.p.) and perfused with the same solution as used for pERK immunohistochemistry.

The medulla and upper cervical spinal cord were removed and post-fixed in 4% PFA for 3 days at 4°C. The tissues were then transferred to 20% sucrose (w/v) in 0.01 M phosphate-buffered saline (PBS) for several days for cryoprotection. Thirty-micron-thick sections were cut with a freezing microtome and every fourth section was collected in 0.01 M PBS. Free-floating tissue sections were rinsed in 0.01 M PBS, 3% normal goat serum in PBS for 1.5 h, and then incubated in rabbit anti-phospho-p44/42 MAP kinase (Thr202/Tyr204) antibody (1∶1000, Cell signaling Technology, Beverly, MA) or rabbit anti-GFAP polyclonal antibody (1∶1000, Dako, Glostrup Denmark) or rabbit anti-Iba1 antibody (1∶1000, Wako, Tokyo, Japan) for 3 days at 4°C. Next, the sections were incubated in biotinylated goat anti-rabbit IgG (1∶600; Vector Labs, Burlingame, CA, USA) for 2 h at room temperature. After washing, the sections were incubated in peroxidase-conjugated avidin-biotin complex (1∶100; ABC, Vector Labs) for 1 h at room temperature. They were then washed in 0.05 M tris buffer (TB), and next incubated in 0.035% 3,3′-diaminobenzidine-tetra HCl (DAB, Tokyo Chemical Industry, Tokyo, Japan), 0.2% nickel ammonium sulfate, and 0.009% peroxide in 0.05 M TB (pH 7.4). The sections were then washed in 0.01 M PBS, serially mounted on gelatin-coated slides, dehydrated in a series of alcohols (from 50 to 100%) and cover slipped.

The pERK- immunoreactive (pERK-IR) cells were drawn under a light microscope with an attached camera lucida drawing tube (Neurolucida 2000 MicroBrightField, Colchester. UT, U.S.A). The number of all pERK-IR cells in Vc was counted from 3 sections of every 6^th^ section and the mean number of pERK-IR cells (/3 sections /rat) was calculated from each animal, in order to reduce the variability of the number of immunoreactive neurons in each section. For GFAP and Iba1 immunohistochemical analyses, the square grid area (26.67×26.67 µm^2^) occupied by the GFAP- and Iba1-immunoproducts in Vc was measured by using a computer-assisted imaging analysis system (Image J 1.37v, NIH, Bethesda, Maryland).

Double immunofluorescence histochemistry was also used to determine if the pERK-IR cells expressed a neuronal label (NeuN), GFAP or Iba1. ION-CCI rats received high-intensity mechanical stimulation (60 g) of the maxillary whisker pad skin (1 Hz, duration for 15 min) and 5 min later were perfused. Thirty-micron-thick sections of the caudal medulla and upper cervical spinal cord were cut with a freezing microtome and processed for double-labeling immunohistochemistry for pERK and NeuN, pERK and GFAP or pERK and Iba1 in the Vc area receiving afferent input from the 2^nd^ branch of the trigeminal nerve [33]. Free-floating tissue sections were rinsed in 0.01 M PBS, 3% normal goat serum in 0.01 M PBS for 1.5 h, and then incubated in rabbit anti-phospho-p44/42 MAPK antibody (1∶300) and mouse anti-NeuN antibody (1∶1000; Chemicon, Temecula, CA), or mouse anti-phospho-p44/42 MAP kinase antibody (1∶200, Cell signaling Technology, Beverly, MA) and rabbit anti-Iba1 antibody (1∶1000) or rabbit anti-GFAP polyclonal antibody (1∶5000) 3 days at 4°C and secondary antibodies (anti-rabbit Arexa Fluor 488 IgG and anti-mouse Arexa Fluor 568, 1∶1000; Invitrogen, Eugene, OR) conjugated for 2 h at room temperature in a dark room. Then the sections were washed in 0.01 M PBS 3 times for 5 min. Sections were mounted on slides and cover-slipped in PermaFluor (Thermo scientific, Fremont, CA).

### PD98059 administration

We used PD98059 as the MEK1 inhibitor to test its effect on the nocifensive behavior, pERK-IR, GFAP-IR or Iba1-IR cells, and Vc neuronal activities in ION-CCI or naïve rats; the solution with 10% DMSO and isotonic saline was used as the vehicle control. PD98059 was dissolved with 10% DMSO and isotonic saline (0.05 µg/ml or 0.1 µg/µl of PD98059) for i.t. administration for 8 days. One day before the ION-CCI rats were anesthetized with pentobarbital Na (50 mg/kg, i.p.) and the C7 spinal process was removed. The silicone tube was inserted into the subdural space from the C7 spinal cord level, and the tip of the silicone tube was placed at the C1/C2 level. The silicone tube was connected with the osmotic pressure pump embedded under the dorsal skin for continuous administration of PD98059. The location of the tip of the tube was determined at the end of the experiment.

### Single neuron recording from Vc

Mechanical head-withdrawal threshold was measured in each rat on day 7 after ION-CCI or sham operation, and the ION-CCI rats showing neuropathic pain behavior and sham rats without neuropathic pain behavior were used for recordings of activity from the Vc (ION-CCI: n = 13, sham: n = 14, ION-CCI+PD98059: n = 8, ION-CCI+vehicle: n = 8). Each group of rats was anesthetized with pentobarbital sodium (50 mg/kg, i.p.) and the trachea and left jugular veins were cannulated to allow artificial respiration and intravenous administration of drugs. Anesthesia was maintained with isoflurane (1–2%) mixed with oxygen during surgery. The rats were mounted in a stereotaxic frame, the medulla was exposed, and a mineral oil pool was made with the skin flaps surrounding the laminectomy. A head holder was rigidly secured to the skull by stainless-steel screws and dental acrylic resin, and the ear bars and nose holder were removed to access to the orofacial region conveniently.

After surgery, anesthesia was maintained throughout the experiment by continuous inhalation of isoflurane (1–2%) mixed with oxygen. During recording sessions, the rats were immobilized with pancuronium bromide (1 mg/kg/h, i.v.) and ventilated artificially. The expired CO_2_ concentration was monitored and maintained between 3.0–4.0%. Rectal temperature was maintained at 37–38°C by a thermostatically-controlled heating pad (Nihon kohden, Tokyo, Japan) and the electrocardiogram was monitored. When the heart beat rate increased more than 300/min, the isoflurane concentration increased appropriately.

Electrophysiological recording procedures were similar to those previously described in detail [11]. Briefly, enamel-coated tungsten microelectrodes (impedance = 10–12 Megohms, 1000 Hz) were advanced into the Vc about 2 mm caudal to the obex in 1 µm steps. Vc neurons were searched by applying mechanical stimulation (pressure or brush) to the craniofacial region. When a single neuron was isolated, its response to mechanical stimulation of the facial skin was carefully examined and its cutaneous receptive field (RF) was mapped. Graded mechanical stimuli were applied to the most sensitive area of the RF. Mechanical stimuli consisted of brushing with a camel hair brush, graded pressure produced by von Frey filaments (1, 6, 15, 26 and 60 g) and pinch produced by a small arterial clip. In order to avoid sensitization due to repeated stimulation, noxious mechanical stimuli were applied to only small area of the RF of each neuron. If the non-noxious RF of the first and second encountered nociceptive neurons overlapped with each other, the second neuron was not included in the analysis. Each neuron was classified either as 1) a low-threshold mechanoreceptive (LTM) neuron; 2) a wide dynamic range (WDR) neuron; or 3) a nociceptive-specific (NS) neuron. In this study only WDR neurons were further studied and their data analyzed. When a WDR neuron was identified, heat and cold stimuli were applied to the most sensitive area of the mechanical RF by a contact thermal probe (5 mm in diameter, adapting temperature: 35°C, Intercross Tokyo Japan). If the neuron responded to cooling of the RF, it was then tested with heat stimulation. The tip of the thermal probe was 5 mm in diameter, and the rate of temperature change was set at 10°C/s. Before application of the thermal stimulus to the RF, the surface temperature was adapted to 35°C for 180 s. Skin heating ranged from 44 to 50°C and lasted for 10 s. Cold stimuli consisted of cooling of the skin from 10 to 30°C. The thermal stimuli were applied to the RF at 190-s intervals (adaptation time: 180 s, stimulus time: 10 s) to avoid sensitization of peripheral nociceptors.

Neuronal responses were fed into a hard disc for subsequent analysis of signals. After recording of the response properties of Vc neurons, lesions were made at the recording site by passing direct current of 20 µA for 15 s. The waveform of single or multiple neuronal activities was analyzed off-line. The waveform of each neuron was identified using Spike 2 software (CED, Cambridge, UK). Peristimulus time histograms (bin width = 1 s) were generated in response to each stimulus. Background activity was first recorded for 10 s before application of mechanical or thermal stimulus and then subtracted from the evoked neuronal responses during the subsequent analysis. Afterdischarges were recorded for 10 s after pinching the RF, and expressed as mean spike frequency. The mean firing frequency was calculated during mechanical or thermal stimulation, and stimulus-response (S-R) functions of each Vc neuron were obtained in response to the mechanical or thermal stimuli. The mechanical or thermal stimulation of the RF was considered to have induced an effect when the peak firing frequency at 5 s after mechanical and 30 s (one trial for each neuron with 180 s intervals) after thermal stimulation differed from the mean background discharge rate by ±2 S.D. The RF of each neuron was drawn to scale on a standard diagram of the rat head. RF area was calculated using image analysis software (Image J 1.37v).

At the end of the experiment, the rats were overdosed with pentobarbital sodium (80 mg/kg, i.p.) and sacrificed. The brains were removed and placed in cold fixative (4% PFA in 0.01 M PBS) for a few days, then transferred to cold phosphate-buffered 20% sucrose for 48 h. Serial sections (50 µm-thick) were cut along the path of the electrode penetration. The sections were counterstained with Thionin for identification of recording sites. Recording sites were drawn using Camera lucida drawing tube (Neurolucida 2000).

### Statistical analysis

Mechanical head-withdrawal thresholds are presented as median values and other data are shown as mean ± SEM. The one-way ANOVA followed by Bonferroni test was performed on the behavioral test at each time point after the operation. Student t-test was used to compare the number of pERK-IR cells between ION-CCI and sham rats, to compare the number of pERK-IR cells between PD98059-injected and vehicle-injected ION-CCI rats, and to compare the heat and cold nocifensive behavior between PD98059-injected and vehicle-injected ION-CCI rats. Student t-test was also used to compare the background activity, afterdischarge, brush and pinch evoked responses between ION-CCI and sham rats, and to compare between PD98059-injected and vehicle-injected ION-CCI rats. For comparison of mechanical nocifensive behavior between PD98059-injected and vehicle-injected ION-CCI rats, Mann Whitney U-test was used. Two-way repeated measures ANOVA followed by Turky test was used to compare the firing frequency following graded mechanical, heat or cold stimuli between ION-CCI rats and sham rats, and to compare between PD98059-injected ION-CCI rats and vehicle-injected ION-CCI rats. Differences were considered significant at p<0.05.

## Results

### Behavioral responses to mechanical and thermal stimulation of the whisker pad skin

**Figure 1:**
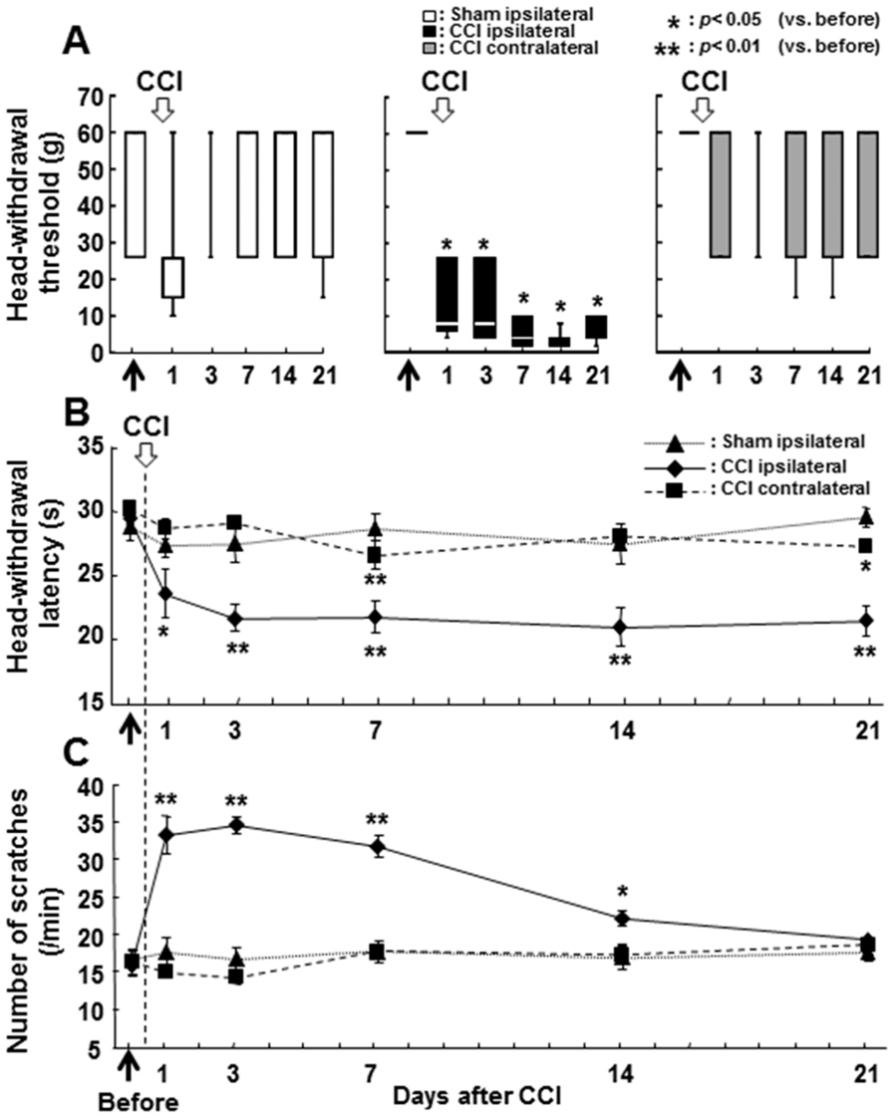
Nocifensive behavior to mechanical, heat or cold stimulation of the whisker pad skin in sham and ION-CCI rats. Time-course change in the head-withdrawal threshold to mechanical stimulation of the whisker pad skin (A), head-withdrawal latency to heat stimulation of the whisker pad skin (B) and face scratching frequency following acetone application to the whisker pad skin (C). Ipsi. to CCI: ipsilateral side to CCI, Contra. to CCI: contralateral side to CCI, Ipsi. to sham: ipsilateral side to sham operation, before: before ION-CCI or sham operation. *: p<0.05 (vs. before), **: p<0.01 (vs. before).

After completion of the training, in which the rats accepted the mechanical stimulation applied to the whisker pad skin, rats underwent the ION-CCI or sham operation. During mechanical or thermal stimulation of the whisker pad skin, vocalization and autotomy were never observed in ION-CCI and sham rats. Figure 1 illustrates the head-withdrawal threshold to mechanical stimulation of the whisker pad skin (A), head-withdrawal latency to heat stimulation of the whisker pad skin (B), and the number of face scratches following cold (acetone) stimulation of the whisker pad skin (C). The head-withdrawal threshold to mechanical stimulation and head-withdrawal latency to heat stimulation of the whisker pad skin were significantly reduced at 1 day after ION-CCI and lasted for 21 days after the CCI compared to that before CCI operation (Fig. 1A and B). The number of face scratches after acetone application to the whisker pad skin was also significantly larger in ION-CCI rats for 14 days after CCI compared to that before the operation. There were no significant changes in head-withdrawal threshold to mechanical stimulation of the whisker pad skin, head-withdrawal latency to heat stimulation, and the number of face scratches after acetone application to the whisker pad skin on the side contralateral to the ION-CCI. In sham rats, the head-withdrawal threshold to mechanical stimulation of the whisker pad skin, the head-withdrawal latency to heat and the number of face scratches after acetone application were also not changed after the operation.

### pERK-IR, GFAP-IR and Iba1-IR cells in Vc

**Figure 2:**
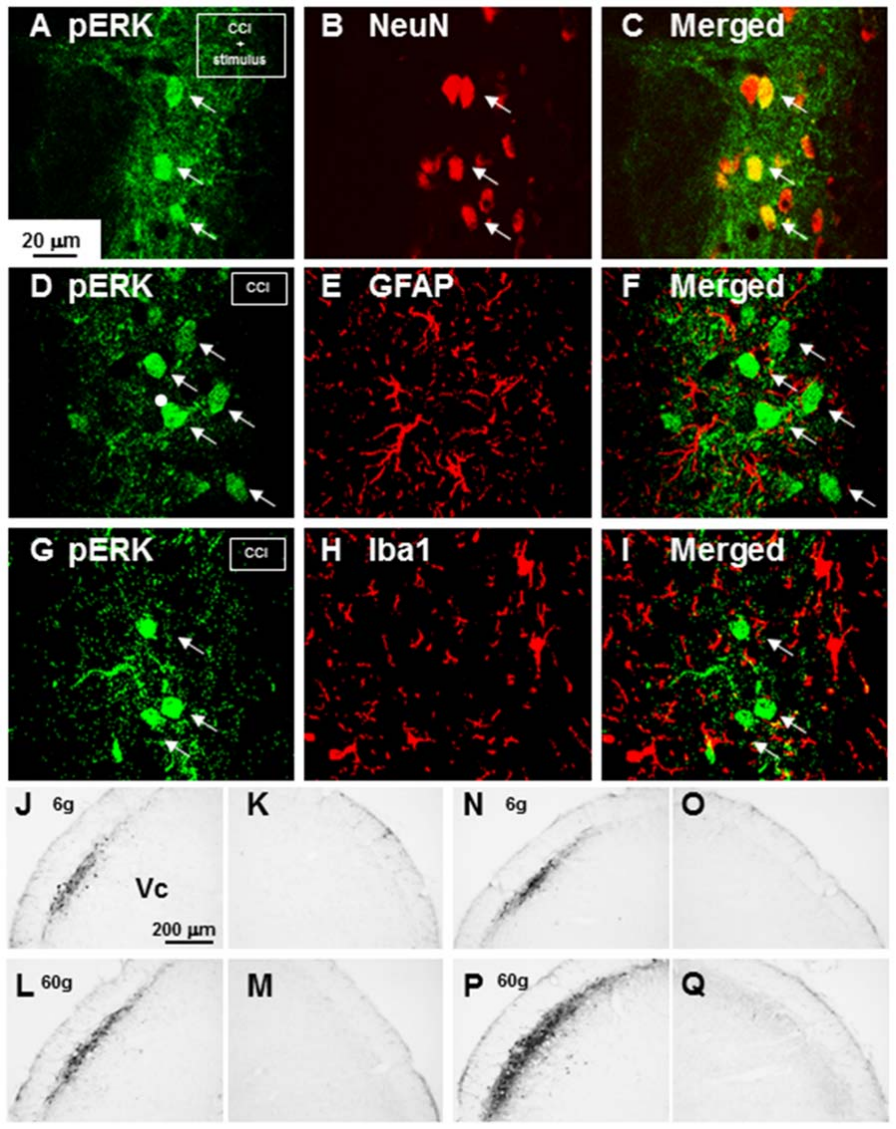
Photomicrographs of pERK-IR, NeuN-IR, GFAP-IR and Iba1-IR cells in Vc. A–C: Fluorescent photomicrographs of pERK-IR cells (A), NeuN-IR cells (B) and merged photomicrograph of A and B (C) ION-CCI rats. D–F: Fluorescent photomicrographs of pERK-IR cells (D), GFAP-IR cells (E) and merged photomicrograph of D and E (F) in ION-CCI rats. G–I: Fluorescent photomicrographs of pERK-IR cells (G), Iba1-IR cells (H) and merged photomicrograph of G and H (I) in ION-CCI rats. J–M: pERK-IR cells following 6 g stimulation of the whisker pad skin on the side ipsilateral to the sham operation (J), those in the contralateral side (K), those following 60 g stimulation on the ipsilateral side (L) and those in the contralateral side (M), N–Q: pERK-IR cells following 6 g stimulation of the whisker pad skin on the side ipsilateral to the ION-CCI (N), those in the contralateral side (O), those following 60 g stimulation in the ipsilateral side (P) and those in the contralateral side (Q).

All pERK-IR cells following noxious mechanical stimulation of the whisker pad skin also showed NeuN immunoreactivity in ION –CCI rats, indicating that pERK-IR cells observed were neurons (Fig. 2 A, B and C). To test if ERK phosphorylation occurs in astroglial or microglial cells in Vc, we studied pERK, GFAP and Iba1 immunohistochemistries in the Vc of ION-CCI rats. No pERK-IR cells showed GFAP-IR (Fig. 2D, E and F) or Iba1-IR (Fig. 2G, H and I) in Vc.

**Figure 3:**
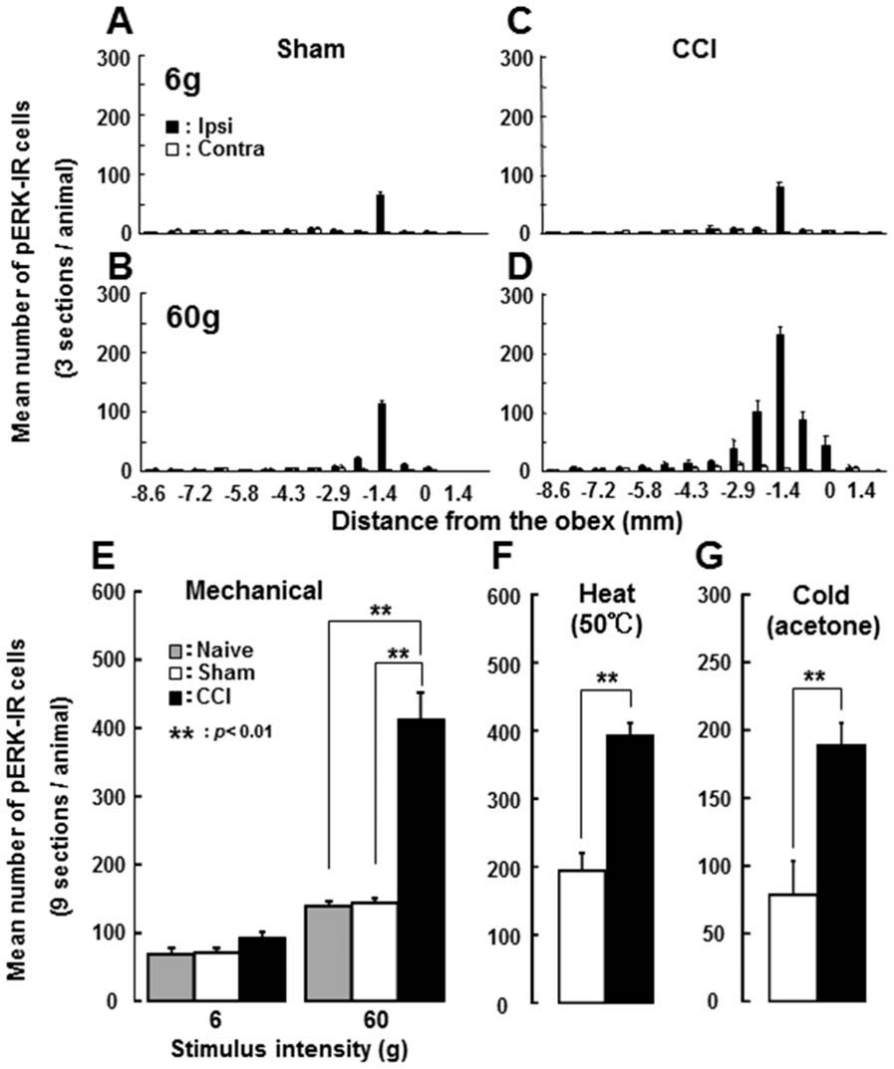
Rostro-caudal distribution and mean number of pERK-IR cells. A and B: rostro-caudal distribution of pERK-IR cells following 6 g (A) and 60 g (B) mechanical stimulation of the whisker pad skin in sham rats. C and D: rostro-caudal distribution of pERK-IR cells following 6 g (C) and 60 g (D) mechanical stimulation of the whisker pad skin in ION-CCI rats. E: Mean number of pERK-IR cells following 6 or 60 g stimulation of the whisker pad skin in naïve, sham and ION-CCI rats. **: p<0.001 (sham vs. ION-CCI).

A large number of pERK-IR cells were observed in the superficial laminae of the Vc on the side ipsilateral to the operation 5 min after non-noxious (6 g) and especially noxious (60 g) mechanical stimulation of the whisker pad skin in sham rats (Figs. 2 J, L and 3 A, B, E) and ION-CCI rats (Figs. 2 N, P and 3 C, D, E) on day 7 after the operation, with the largest number at 1.4 mm caudal to the obex (Fig. 3 A–D). No pERK-IR cells were observed in the contralateral Vc of sham rats (Fig. 2 K and M) and ION-CCI rats (Fig. 2 O and Q). The mean number of pERK-IR cells after noxious stimulation (60 g) of the whisker pad skin was significantly larger in ION-CCI rats compared to sham rats on the side ipsilateral to the operation, whereas no significant difference in the number of pERK-IR cells between sham and ION-CCI rats was observed after non-noxious mechanical stimulation (6 g) of the whisker pad skin (Fig. 3 E). The number of pERK-IR cells following heat (50°C) or cold (acetone administration) stimulation was also significantly larger in ION-CCI rats compared to sham rats (Fig. 3 F and G).

### Responses of Vc nociceptive neurons

**Figure 4:**
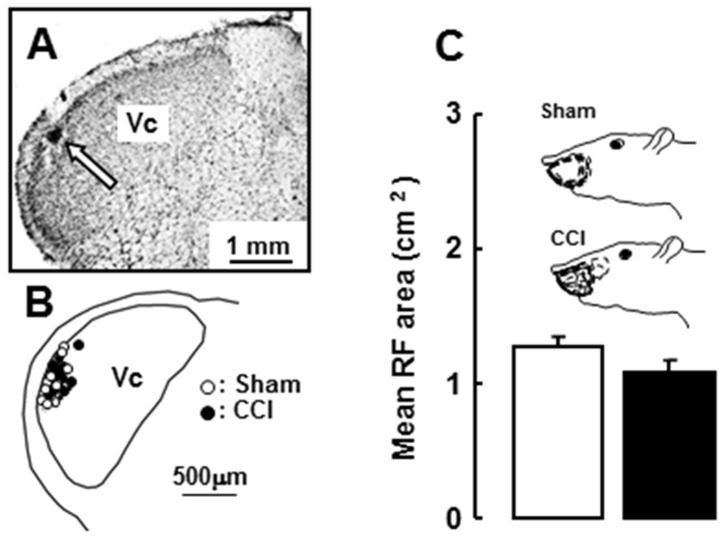
Recording sites and receptive field size. A and B: location of recording sites in Vc. C: Drawings of RFs and mean RF area in ION-CCI and sham rats.

**Figure 5:**
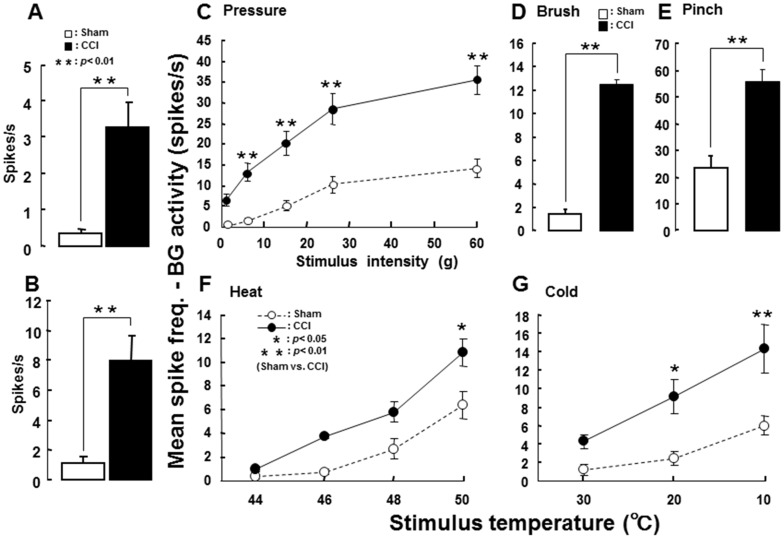
Single neuronal activity of Vc WDR neurons in ION-CCI and sham rats. A: Mean background activities. B: Mean afterdischarges. C: Mean spike frequency following graded mechanical stimuli. D: Mean spikes following brushing to the RF. E: Mean spikes following pinching to the RF. F: Mean spike frequency following graded heat stimuli. G: Mean spike frequency following graded cold stimuli. *: p<0.05 (sham vs. ION-CCI), **: p<0.01 (sham vs. ION-CCI).

A total of 27 nociceptive neurons was recorded from Vc in sham and ION-CCI rats (sham: 14, ION-CCI: 13), and electrophysiological properties of functionally identified Vc nociceptive neurons were analyzed. Most of these nociceptive neurons in sham and ION-CCI rats were located in the superficial laminae of the lateral portion of the Vc (Fig. 4 A and B). The nociceptive neurons were defined as WDR neurons based on their responses to mechanical stimulation of their RFs. The RF of each WDR neuron was located on the whisker pad skin, and the mean RF size was not different between sham and ION-CCI rats (Fig. 4 C). Mean background activity and afterdischarge of WDR neurons were significantly larger in ION-CCI rats compared to sham rats (Fig. 5 A and B). WDR neurons showed graded responses to graded stimulation of the RF in sham and ION-CCI rats (Fig. 5 C), and the mean graded pressure responses and brush- and pinch-evoked responses were significantly larger in ION-CCI rats compared to sham rats (Fig. 5 D and E). Heat and cold-evoked responses were also significantly larger in ION-CCI rats compared to sham rats (Fig. 5 F and G).

### Effect of i.t. PD98059 administration on ERK phosphorylation in Vc neurons, nocifensive behavior, Vc WDR neuronal activities, GFAP-IR cells and Iba1-IR cells

**Figure 6:**
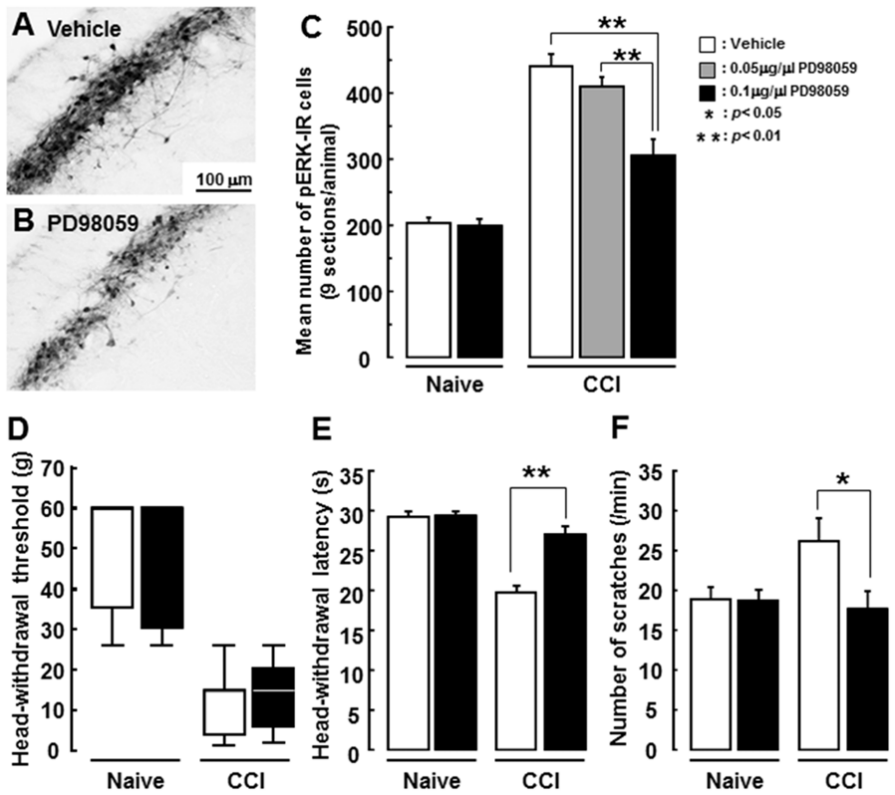
Effect of i.t. administration of PD98059 on ERK phosphorylation in Vc neurons and nocifensive behavior. A and B: photomicrographs of pERK-IR cells in Vc following 60 g stimulation of the whisker pad skin in ION-CCI rats with i.t. vehicle (A) or PD98059 (B) administration. C: Mean number of pERK-IR cells in naïve and ION-CCI rats with vehicle or PD98059 administration. D–F: Mean head-withdrawal threshold to mechanical stimulation of the whisker pad skin (D), mean head-withdrawal latency to heat stimulation of the whisker pad skin (E) and mean number of face scratches after acetone application to the whisker pad skin (F) in naïve and ION-CCI rats with vehicle or PD98059 administration. *: p<0.05, **: p<0.01 (vehicle vs. PD98059).

Many pERK-IR cells after noxious mechanical stimulation (60 g) of the whisker pad skin were apparent in the superficial laminae of the Vc in ION-CCI rats after i.t. vehicle injection (Fig. 6 A), but the mean number of pERK-IR cells was significantly smaller in 0.1 µg/µl concentration of PD98059-injected ION-CCI rats compared to vehicle-injected ION-CCI rats after 60 g stimulation of the whisker pad skin (Fig. 6 C). We also tested the effect of 0.05 µg/µl concentration of PD98059 on ERK phosphorylation in Vc neurons. The number of pERK-IR cells was slightly smaller than saline-injected rats but not reached to the significant level (Fig. 6C). In naïve rats, we could not observe significant effect of PD98059 administration on pERK expression and nocifensive behaviors. The mean head-withdrawal threshold to mechanical stimulation of the whisker pad skin was not different between PD98059-injected and vehicle-injected ION-CCI rats (Fig. 6 D). On the other hand, mean head-withdrawal latency to heat stimulation of the whisker pad skin was significantly longer in PD98059-injected ION-CCI rats compared to vehicle-injected ION-CCI rats (Fig. 6 E), and the mean number of face scratches after acetone administration to the whisker pad skin was also significantly smaller in PD98059-injected ION-CCI rats compared to vehicle-injected ION-CCI rats (Fig. 6 F).

**Figure 7:**
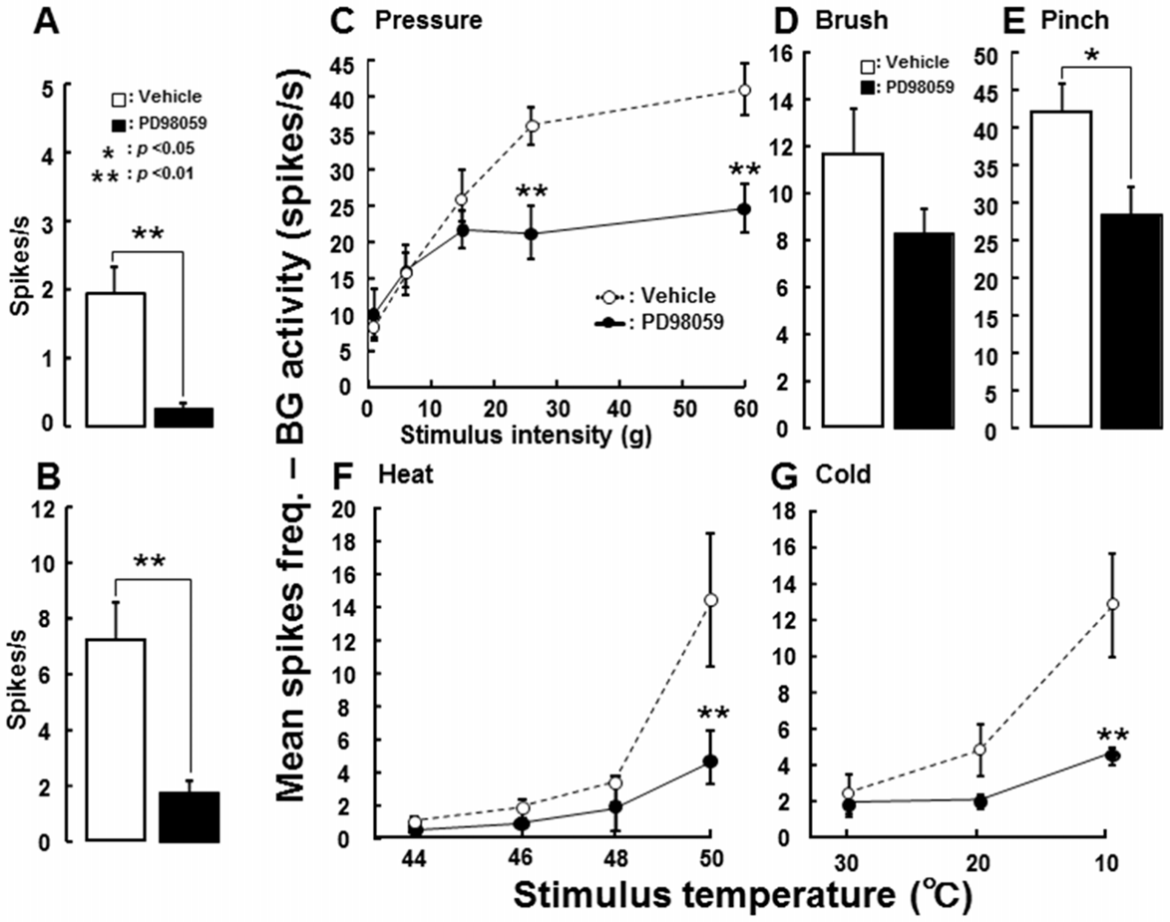
Effect of i.t. administration of PD98059 on Vc WDR neuronal activities in ION-CCI rats. A: Mean background activities. B: Mean afterdischarges. C: Mean spike frequency following graded mechanical stimuli. D: Mean spikes following brushing the RF. E: Mean spikes following pinching the RF. F: Mean spike frequency following graded heat stimuli. G: Mean spike frequency following graded cold stimuli. *: p<0.05, **: p<0.01 (vehicle vs. PD98059).

We also studied the effect of i.t. administration of PD98059 on Vc WDR neuronal activities in ION-CCI rats. A total of 16 WDR neurons was tested with i.t. administration of vehicle or PD98059 in ION-CCI rats (PD98059: n = 8, vehicle: n = 8). Mean background activity and afterdischarge in Vc WDR neurons were significantly smaller in PD98059-administered ION-CCI rats compared to vehicle-administered ION-CCI rats (Fig. 7 A and B). Evoked responses of Vc WDR neurons following noxious (60 g) but not non-noxious mechanical stimulation (less than 15 g) of the whisker pad skin were significantly smaller in PD98059-administered ION-CCI rats compared to vehicle-administered ION-CCI rats (Fig. 7 C). Responses to brushing of the RF were not affected by PD98059 administration in ION-CCI rats (Fig. 7 D), whereas responses to pinching of the RF were significantly depressed after PD98059 administration (Fig. 7 E). Noxious heat (50°C) or cold (10°C)-evoked responses of Vc WDR neurons were also significantly suppressed in ION-CCI rats after i.t. administration of PD98059 (Fig. 7 F and G).

**Figure 8:**
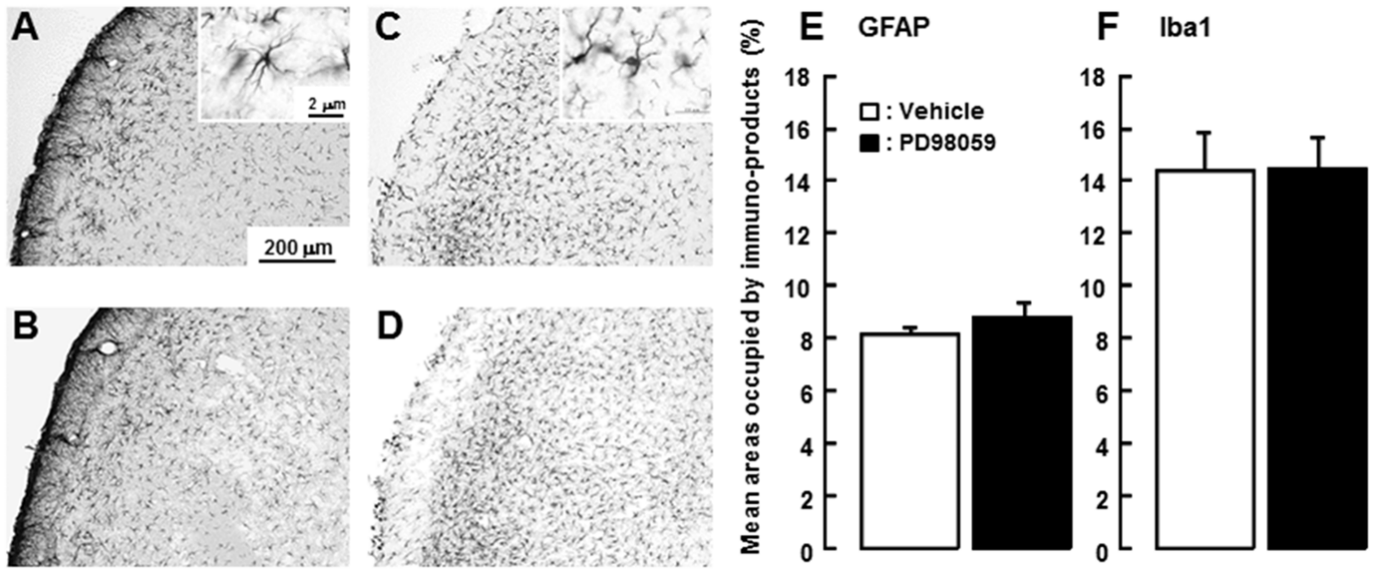
Effect of PD98059 administration on GFAP and Iba1 immunoreactivities in ION-CCI rats. A and B: Photomicrographs of GFAP-IR cells in Vc in ION-CCI rats with i.t. PD98059 (A) or vehicle (B) administration. C and D: Photomicrographs of Iba1-IR cells in Vc in ION-CCI rats with i.t. PD98059(C) or vehicle (D) administration. E and F: Mean areas occupied by GFAP-IR (E) and Iba1-IR (F) immuno-products in ION-CCI rats with i.t. vehicle or PD98059 administration.

To evaluate the effect of i.t. administration of PD98059 on glial cell hyperactivation in Vc of ION-CCI rats (PD98059: n = 5, vehicle: n = 5), GFAP and Iba1 immunoreactive cells were studied in ION-CCI rats following i.t. administration of PD98059 or vehicle. Many GFAP-IR (Fig. 8 A and B) or Iba1-IR cells (Fig. 8 C and D) were observed in Vc of ION-CCI rats with PD98059 or vehicle administration, and the areas occupied by GFAP-IR and Iba1-IR immuno-products in Vc were not significantly different between PD98059-administered and vehicle-administered ION-CCI rats (Fig. 8 E and F).

## Discussion

The present study has provided novel findings of the effects of ION-CCI on the rat, documenting mechanical or thermal behavioral hypersensitivity of the whisker pad skin innervated by the injured ION, significant increases in the number of pERK-IR cells in the Vc following noxious but not non-noxious stimulation of the whisker pad skin, and changes in the response properties of Vc WDR neurons that are indicative of Vc central sensitization. Further novel findings were that the thermal but not mechanical behavioral hypersensitivity, the number of pERK-IR cells and responses of Vc WDR neurons to noxious mechanical or thermal stimuli but not non-noxious stimuli could be significantly suppressed after i.t. injection of MEK1 inhibitor in ION-CCI rats, suggesting that ERK phosphorylation in Vc WDR neurons may contribute to thermal but not mechanical hypersensitivity in the face following trigeminal nerve injury.

### Mechanical and thermal hypersensitivity following ION-CCI

ION-CCI produced sustained modifications in sensory processing, resulting in long-lasting mechanical and heat hypersensitivity and transient hypersensitivity to cold stimulation in the area innervated by the injured ION. In our ION-CCI rats, mechanical and heat hypersensitivity was obvious at 1 day after ION ligation and lasted for at least 21 days, whereas cold hypersensitivity lasted only 14 days although it also had an early onset. The hypersensitivity to mechanical and heat stimulation to the hind paw in rats with sciatic nerve ligation has been reported to be apparent at 2 days post-injury and to last for more than 30 days [34], [35], and cold hypersensitivity of the hind paw has also been reported in rats following sciatic nerve ligation to last for more than 21 days at the early onset (1 day after the ligation) [36]. Thus, sciatic or trigeminal nerve injury induces hind paw or face mechanical and heat hypersensitivity with an early onset that persists for a long period, whereas cold hypersensitivity lasts for short period following trigeminal nerve injury and persists for a long period after sciatic nerve injury.

### Changes in Vc neuronal activity associated with mechanical and thermal hypersensitivity

It has been reported that the behavioral hypersensitivity to innocuous mechanical or thermal stimulation following spinal nerve injury originates from increased neuronal excitability in the peripheral and central nervous system [37], [38], [39]. In the case of peripheral changes, numerous studies have shown that primary afferent fibers have significantly higher spontaneous activity following spinal nerve injury [40], [41], [42], and sensitization of the injured primary afferent fibers may occur [17], [41], [43]. A number of papers have also described altered electrophysiological properties and protein production in myelinated and unmyelinated fibers in sciatic nerve-ligated rats [43], [44], [45], [46] and that TRPM8 and TRPV1 channel expression is significantly enhanced in DRG neurons after sciatic nerve injury [47]. In the orofacial region, ION-CCI and IAN-transection models have been developed to evaluate mechanisms underlying orofacial neuropathic pain [6], [11], [12], [13], [14]. Molecular changes and enhancement of neuronal excitability can be observed in TG neurons [15], [16], [17] and Vc neurons after infraorbital or cervical nerve injury [18], [19], [20]. These findings suggest that the mechanical and thermal hypersensitivity that we documented after ION-CCI may be associated with hyperexcitability of injured myelinated or unmyelinated ION primary afferent fibers. It is noteworthy that the heat and mechanical hypersensitivity lasted for at least 21 days after the ION-CCI, whereas cold hypersensitivity lasted for 14 days after ION-CCI. It is possible that the different time-courses in nocifensive behaviors between mechanical or heat hypersensitivity and cold hypersensitivity in the ION-CCI rats may be due, at least in part, to different effects of ION-CCI on mechanical or heat sensitive primary afferent fibers versus cold-sensitive fibers, such as channel or receptor expression in each type of primary afferent neurons after the CCI [17].

The possibility of central nervous mechanisms contributing to the above differences also cannot be ruled out. Spinal dorsal horn (DH) and thalamic nociceptive neurons have high background activity and enhancement of mechanical and/or thermal evoked responses following peripheral nerve injury [4], [37], [48]. Moreover, both non-noxious and noxious responses may be enhanced after spinal nerve injury, indicating that these CNS neuronal changes may contribute to the mechanical and thermal hypersensitivity is produced by peripheral nerve injury [34], [37]. In the trigeminal system, it has also been reported that non-noxious and noxious responses of Vc neurons as well as IAN fibers are enhanced following IAN injury [6], [32], [49]. The ION-CCI-induced increase in Vc neuronal excitability documented in this study reflected changes in Vc neuronal properties indicative of central sensitization of Vc nociceptive neurons and were similar to those reported in these previous studies, in that background activity, afterdischarges and mechanical and thermal-evoked responses were significantly enhanced in IAN-injured rats compared to sham rats. These findings suggest that these Vc neuronal changes likely contributed to the mechanical, heat and cold hypersensitivity in the face following ION-CCI. We also observed a significant increase in the number of pERK-IR cells double-labeled with NeuN following noxious but not non-noxious mechanical stimulation of the whisker pad skin in ION-CCI rats compared to sham rats. This observation suggests that the hyperexcitability of Vc nociceptive neurons is associated with an enhancement of ERK phosphorylation in Vc nociceptive neurons following noxious but not non-noxious mechanical stimulation of the whisker pad skin in ION-CCI rats.

### Functional implications of ERK phosphorylation in trigeminal neuropathic pain

We also studied the effect of the MEK1 inhibitor PD98095 on the head-withdrawal behavior to mechanical or heat stimulation of the whisker pad skin and on face scratching behavior evoked by acetone application to the whisker pad skin in ION-CCI rats. The head-withdrawal latency to heat stimulation and face scratching frequency to acetone application were significantly reduced in the ION-CCI rats following i.t. administration of PD98059, whereas the head-withdrawal threshold to mechanical stimulation of the whisker pad skin was not affected by the PD98059 administration. We also demonstrated that Vc neuronal responses evoked by noxious heat, cold and noxious mechanical stimulation but not by non-noxious mechanical stimulation were significantly suppressed following i.t. PD98059 administration compared to vehicle administration in the ION-CCI rats. Non-noxious stimulus which was almost same intensity as mechanical head-withdrawal threshold causes ERK phosphorylation in the small number of Vc and C1–C2 neurons in ION-CCI rats. Furthermore, the number of pERK-IR cells following non-noxious mechanical stimulation in ION-CCI rats was not significantly larger than that of sham rats, and we could not observe significant effect of PD98059 on the excitability of WDR neurons in IN-CCI rats. These results suggest that pERK-IR cells are involved in noxious thermal and/or noxious mechanical responses of Vc and C1–C2 neurons but not non-noxious mechanical responses. Furthermore, the number of pERK-IR cells ION-CCI rats was not significantly larger in sham rats following non-noxious mechanical stimulation of the lateral facial skin. The head-withdrawal threshold and non-noxious responses of WDR neurons were not affected by PD98059 administration. These data are consistent with our behavioral data, in which head-withdrawal responses to non-noxious mechanical stimulation of the whisker pad skin was not affected by PD98059 administration in ION-CCI and sham rats. In addition, the increased number of pERK-IR cells following noxious stimuli in ION-CCI rats could be significantly reduced by PD98059 administration. There is evidence that ERK phosphorylation is involved in the enhanced neuronal activity in the spinal DH following strong noxious stimuli, injury or inflammation since the increased firing of DH neurons induced by repetitive electrical stimulation of the sciatic nerve at C-fiber intensity can be significantly suppressed following i.t. administration of the MEK1 inhibitor U0126 [50], [51], [52], [53], [54], [55], and significant enhancement of pERK expression occurs in DH nociceptive neurons following peripheral nerve injury or inflammation that also can be suppressed by MEK1 inhibitor administration [27], [56], [57], [58]. We have also documented increased pERK-IR cells in Vc following other types of noxious stimuli [18], [28], [30], [31]. Together with these previous data, the findings suggest that ERK phosphorylation is involved in the enhancement of the Vc nociceptive neuronal activity in ION-CCI rats that results from activation of small-diameter primary afferent fibers by strong noxious mechanical or thermal stimulation.

Since some previous studies have documented that ERK phosphorylation occurs in glial cells in rats with sciatic nerve injury or spinal nerve ligation [59], [60], we also tested if i.t. administration of PD98059 in ION-CCI rats affects expression of GFAP and Iba1 that are markers for astroglial and microglial cells, respectively. We did not observe any differences in the areas occupied by GFAP and Iba1 immuno-products between PD98059- and vehicle-administered ION-CCI rats. There was also no evidence of any pERK-IR cells showing GFAP and Iba1 immunoreactivities in ION-CCI rats, but all pERK-IR cells showed NeuN immunoreactivity. These findings suggest that ERK may be phosphorylated in neurons but not in astroglial or microglial cells in Vc in this neuropathic pain model.

Taken together with previous results, the present findings suggest that ERK phosphorylation in Vc neurons is involved in central sensitization of Vc nociceptive neurons following trigeminal nerve injury, resulting in orofacial thermal hypersensitivity associated with trigeminal neuropathic pain.
